# Nanoscale Morphology, Interfacial Hydrogen Bonding, Confined Crystallization and Greatly Improved Toughness of Polyamide 12/Polyketone Blends

**DOI:** 10.3390/nano8110932

**Published:** 2018-11-08

**Authors:** Siyuan Li, Yan Yang, Xiangjun Zha, Yicun Zhou, Wei Yang, Mingbo Yang

**Affiliations:** State Key Laboratory of Polymer Materials Engineering, College of Polymer Science and Engineering, Sichuan University, Chengdu 610065, China; lisiyuan7899@sohu.com (S.L.); smsyang135@163.com (Y.Y.); zhaxj@foxmail.com (X.Z.); yicunzhou@126.com (Y.Z.)

**Keywords:** nanostructured blends, confined crystallization behavior, PA12/PK blends, toughness

## Abstract

Nanostructured polyamide 12(PA12)/polyketone (PK) blends were fabricated by melt compounding. The nanoscale droplet and domain-in-domain morphologies depending on PK content were observed. When the content of PK was 10 vol %, the impact strength of the blend jumped from 6.8 to 111.9 kJ/m^2^ and further improved with an increasing content of PK. The toughening mechanism was found to be closely related with the morphology change from nanoscale droplet morphology to domain-in-domain morphology owing to the strong interfacial hydrogen bonding. The nanoscale morphology confinement and interfacial hydrogen bonding enhances the crystallization kinetics, while it lowers down the thermodynamic stability of the crystals. The toughening mechanisms were discussed based on these factors.

## 1. Introduction

Constructing nanostructures in polymer materials can lead to high performance because of the fascinating nanoeffect [1,2,3]. In recent years, many studies on polymer nanocomposites introduced various nanofillers into polymers to produce high performance or functional materials [4,5,6,7,8,9,10]. However, some key issues greatly restrict the wide applications of these materials, such as the high expense of nanofillers, bad dispersion of nanofiller in polymers, material processing and reduced mechanical performance, especially toughness. Therefore, nanostructured polymer blends (NPBs) have attracted enormous interest, because such polymer blends can be produced by controlling the phase morphologies of polymer blends at the nanoscale.

However, it is challenging to obtain NPBs due to the thermodynamic instability of polymer phases and domains [11]. Generally, micron-sized domains are created, owing to the phase separation (spinodal or nucleation and growth) of polymer blends. Some special methods and processing techniques have been applied to produce NPBs, such as micro-phase-separation or reaction blending. Using the self-assembling properties of a diblock or triblock copolymer, nanostructured polymer/block copolymer blends can be produced. Bates et al. [12,13] blended poly(ethylene oxide)-*b*-poly(ethyl ethylene) (PEO-PEE) and poly(ethylene oxide)-*b*-poly(ethylene propylene) (PEO-PEP) diblock polymers with epoxy resins. They found that the nano-phase structures were generated through the self-assembly mechanism and that the nano morphology depended on the blend composition. Yang et al. [14] produced a nanostructured epoxy thermosets via reaction-induced phase separation by blending poly(ɛ-caprolactone)-block-poly(butadiene-co-acrylonitrile)-block-poly(ɛ-caprolactone) (PCL-*b*-PBN-*b*-PCL) into epoxy resin. The results showed that this nanostructured blends exhibited high fracture toughness. Todd et al. [15] reported a versatile method of in situ polymerization of macrocyclic carbonates in the presence of a maleic anhydride polypropylene (mPP) matrix and a surface-active compatibilizer to yield micro- and nanostructured polymer blends consisting of a polycarbonate minor phase in polypropylene matrix. Polyamide(PA)/polyethene (PE) [16] and poly(vinylidene fluoride) (PVDF)/poly(3-thiophene methyl acetate) (PTMA) [17] nanostructured blends were also reported to be produced by the reactive blending method. Additionally, there have been many efforts to produce NPBs by using special processing methods such as high shear extrusion molding [18,19]. However, due to the thermodynamic instability and high interfacial tension between phases, the enhancement of mechanical properties achieved is still limited. Nano-sized domains in polymer blends can be stabilized by introducing suitable compatibilizers, which help to decrease the interfacial tension between phases. Kim et al. [20] compared the phase morphologies of polystyrene (PS)/polycaprolactone (PCL) blends compatibilized with four different types of compatibilizers. They found that the PCL domain size could be maintained at a level of about 100 nm by 5 wt % nearly symmetric styrene/4-hydroxystyrene di-block copolymer. Tol et al. [21] reported that styrene-maleic anhydride (SMA) copolymer can be used to control the phase morphology and reduce the size of dispersed PA6 droplets down to 100–150 nm in PS/polyamide 6 (PA6) and PPE/PS/PA6 blends.

It should be pointed out that some polymer pairs can intrinsically form nanostructured blends by using common processing methods such as extrusion compounding. Li et al. [22] found that the binary system of poly(vinylidene fluoride) (PVDF) and acrylic rubber (ACM) can form nanostructured blends. It was shown that the ACM domains could be dispersed in a PVDF matrix with an average domain size less than 100 nm, and the nanodomains affected the crystallization behaviors and mechanical properties of the blends greatly. Banerjee et al. [23] produced a polyamide-based thermoplastic elastomeric blend with a PA6 matrix and nanoscale fluorocarbon elastomer particles. They analyzed the influence of the interactions between the components and morphologies on the physical properties of the blends. Asano et al. [24,25] studied a nanostructured PA6/polyketone (PK) blend and carefully characterized the nanostructure of the PA-rich phase and PK-rich lamellar network. The impact resistance of PA6/PK blends was found to be superior to that of a similar polycarbonate material with a phase-separated structure and higher-order structure. Obviously, the preparation of NPBs with common melt compounding processes was of great importance for industry-scale production and applications. A perquisite is that the selected polymer pairs should exhibit appropriate miscibility [22], and polymers containing certain functional groups, capable of forming specific interactions such as hydrogen bonding, are of particular interest [26]. These polymers include PA, PVDF, PK and poly(methyl methacrylate) (PMMA), with polar –NH, –CF, –CO and ester groups, respectively. Of course, some other key parameters, such as viscosity difference between the components, are also important for the design of NPBs [27].

In this work, a polyamide 12(PA12)/PK blend was selected to be a model system owing to the specific interaction between the amide groups in PA12 and carbonyl groups in PK can hopefully lead to a partially miscible blend. Moreover, the large differences in the melting and crystallization temperature of PA12 and PK made it possible for us to examine the crystallization behavior of the individual polymer component separately. The results demonstrated that nanostructured PA12/PK blends can be produced easily using conventional extrusion molding, and that the blends show greatly improved toughness owing to the nanostructured morphology, interfacial hydrogen bonding and confined crystallization behaviors of the nanoscale domains.

## 2. Materials and Methods

### 2.1. Materials and Sample Preparation

PA12 with a density of 1.01 g/cm^3^ and a melting point of 180 °C, was purchased from Arkema, Columbus, France. The PK used was a commercial-grade resin (M630A, Hyosung Co., Seoul, Korea) with a density of 1.24 g/cm^3^ and a melting point of 220 °C.

PA12 and PK were dried at 80 °C in a vacuum oven for 24 h and the blends were produced using a co-rotating twin-screw extruder (SHJ-20, Gaint Mach., Nanjing, China, the screw diameter is 20 mm). The processing temperatures were 190–240 °C from hopper to die and the rotating speed of the screw was 180× rpm. After melt blending, the samples were compression molded into sheets with a thickness of about 2 mm at 240 °C and 10 MPa for 10 min. The resulted samples were named AxKy, where x and y stand for the volume fraction of PA12 and PK in the blends, respectively. For example, A8K2 means the blend with 80 vol % PA12 and 20 vol % PK.

### 2.2. Characterization

#### 2.2.1. Morphological Observation

The phase morphology was characterized with a JEOL JSM-5900LV scanning electron microscope (SEM, JEOL, Tokyo, Japan), which operated at an accelerating voltage of 20 kV. The samples were immersed in liquid nitrogen for 1 h, and then impact fractured. The dispersed phase domains of PK were etched in pyrrole for 3 h at 40 °C. Before SEM observation, all the fractured surfaces were gold sputtered.

Furthermore, the phase morphologies were also examined with a high-resolution transmission electron microscope (TEM, FEI, Tecnai G2 F20, FEI Company, Hillsboro, OR, USA), equipped with a field emission gun operating at 200 kV. Before observation, the samples were cryomicrotomed into 60 nm-thick sections at −80 °C and treated in phosphotungstic acid for 30 min to selectively stain PA12.

#### 2.2.2. Fourier Transform Infrared Spectroscopy (FTIR)

Fourier Transform Infrared Spectroscopy (FTIR) spectra were recorded using a Nicolet 6700 FTIR spectrometer (Thermo Fisher Scientific Inc., Waltham, MA, USA) from 500 to 4000 cm^−1^ with a resolution of 4 cm^−1^ in attenuated total reflection mode and with an accumulation of 32 scans.

#### 2.2.3. Dynamical Mechanical Analysis (DMA)

Dynamical Mechanical Analysis (DMA) characterization of the neat polymers and blends was performed with a TA Instrument (Model Q800, Milford, MA, USA) in a double cantilever mode. The samples with the dimension of 20 mm × 10 mm × 4 mm were tested at a frequency of 1 Hz and strain amplitude of 15 μm. The heating rate was 3 °C/min over the temperature range of −50 to 120 °C.

#### 2.2.4. Contact Angle Measurements

Contact angles were characterized by a KRÜSS DSA100 (KRÜSS, Hamburg, German) instrument. The measurements were performed at room temperature with water and diiodomethane as solvents in the sessile drop mode. Contact angles were measured on a wetting solvent at room temperature, and the results were the average values of at least five replicates.

Equation (1) for water and Equation (2) for diiodomethane according to Margolina and Wu [28], were used to calculate surface tension, polar and dispersion components:

(1) $$\left(1+\cos\theta_{H_{2}O}\right)\gamma_{H_{2}O}=4\left(\frac{\gamma_{H_{2}O}^{d}\gamma^{d}}{\gamma_{H_{2}O}^{d}+\gamma^{d}}+\frac{\gamma_{H_{2}O}^{p}\gamma^{p}}{\gamma_{H_{2}O}^{p}+\gamma^{p}}\right)$$

(2) $$\left(1+\cos\theta_{CH_{2}I_{2}}\right)\gamma_{CH_{2}I_{2}}=4\left(\frac{\gamma_{CH_{2}I_{2}}^{d}\gamma^{d}}{\gamma_{CH_{2}I_{2}}^{d}+\gamma^{d}}+\frac{\gamma_{CH_{2}I_{2}}^{p}\gamma^{p}}{\gamma_{CH_{2}I_{2}}^{p}+\gamma^{p}}\right)$$

where $\gamma =\gamma^{d}+\gamma^{p}$, $\gamma =\gamma_{H_{2}O}^{d}+\gamma_{H_{2}O}^{p}$, $\gamma =\gamma_{CH_{2}I_{2}}^{d}+\gamma_{CH_{2}I_{2}}^{p}$ and *γ* is the surface tension, *p* is the polar component, *d* is the dispersion component and $\theta_{H_{2}O}$ and $\theta_{CH_{2}I_{2}}$ are the contact angles of the polymers with water and diiodomethane, respectively. Other parameters are $\gamma_{H_{2}O}^{d}$ = 22.1 dyn/cm, $\gamma_{H_{2}O}^{p}$ = 50.7 dyn/cm, $\gamma_{CH_{2}I_{2}}^{d}$ = 44.1 dyn/cm and $\gamma_{CH_{2}I_{2}}^{p}$ = 6.7 dyn/cm [29].

#### 2.2.5. Differential Scanning Calorimetry (DSC)

For the non-isothermal crystallization behavior, the samples were first heated to 250 °C and maintained at 250 °C for 3 min to eliminate the thermal history, followed by a cooling scan down to 40 °C, and a second heating scan up to 250 °C with a differential scanning calorimetry (DSC) Q20 (TA Instruments, Milford, MA, USA) under a nitrogen atmosphere at the flow rate of 50 mL/min. The rate of heating and cooling was 10 °C/min. The normalized crystallinity (*Xc*) of PA12 or PK component was calculated with Equation (3):

*Xc* = (*ΔHm*/*ΔH*^0^*m*)/*α* (3)

where α is the weight ratio of PA12 or PK, *ΔHm* is the melt enthalpy and *ΔH^0^m* is the theoretical melt enthalpy for 100% crystallized sample whose values were taken as 209.2 J/g for PA12 [30] and 227 J/g for PK [31], respectively.

#### 2.2.6. Polarized Optical Microscopy (POM)

Slices from the samples were observed on a polarized optical microscopy (Olympus BX-51, Olympus Tech, Tokyo, Japan) with a Micropublisher RTV 5.0 digital camera (DC, Tokyo, Japan). The samples were melted at 250 °C for 3 min and then cooled to 25 °C at 10 °C/min on a Linkam hot stage CSS450 (Tokyo, Japan). The thickness of the slices was about 20 μm and the captured images at 25 °C after melting were used to inspect the crystalline morphology in the blends.

#### 2.2.7. In Situ Wide Angle X-ray Diffraction (WAXD)

In situ two-dimensional (2D) wide angle X-ray (WAXD) measurements were performed using Rigaku Denki RAD-B diffractometer at Shanghai Synchrontron Radiation Facility (SSRF, Shanghai, China). The samples were 1 mm thick and 1.5 mm wide and the sample-to-detector distance was 110 mm. The wavelength of the monochromatic X-ray was 0.124 nm. The in situ WAXD characterization was carried out with the following procedure. Samples were first heated from 25 to 240 °C at 50 °C/min and maintained at 240 °C for 3 min to erase thermal history. Then, the samples were cooled down at a rate of 10 °C/min to 170 °C, at which PK continues to crystallize, but PA12 is in the melting state. The samples were isothermally crystallized at this temperature for 10 min to complete the crystallization of PK. Subsequently, the samples were cooled down to 25 °C. The WAXD patterns were collected continuously during the process.

#### 2.2.8. Impact Toughness

Rectangular samples (80 mm × 10 mm × 4 mm) were injection molded on a mini-injection molding machine (Thermo Scientific HAAKE Minijet, Waltham, MA, USA). The barrel temperature was set to be 240 °C and the mold temperature was set to be 100 °C. The injection pressure was set to 80 MPa, while holding pressure was set to 50 MPa and the holding time was 10 s. The notched impact strength was tested with a UJ-40 impact testing machine at 25 °C, according to ASTM D256-10. The final values are averages for at least five samples.

## 3. Results and Discussion

### 3.1. Morphology

As has been mentioned, partial miscibility between the components of the blends is of critical importance to produce high-performance NPBs [22]. On the one hand, for two completely miscible polymers, the blend becomes a homogeneous mixture of the two polymers at the molecular scale, and a single-phase material is obtained, which cannot lead to a large improvement of mechanical performance compared to that of the constituting polymers. On the other hand, polymer blends made of completely immiscible polymers give poor mechanical performance owing to extremely poor interfacial adhesion between the two phases and unfavorable morphologies driven by a strong tendency for phase separation.

**Figure 1:**
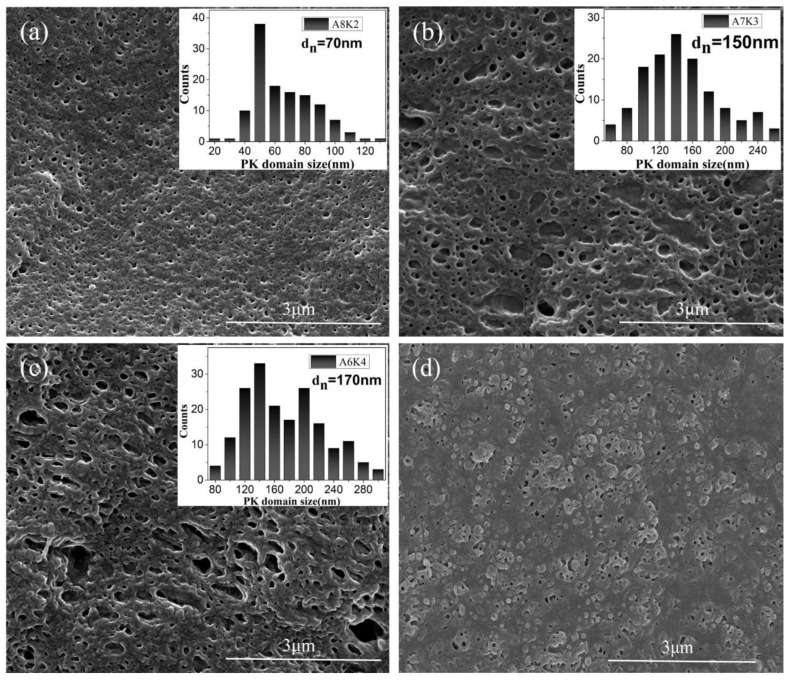
Scanning electron microscope (SEM) images of nanostructured polyamide 12 (PA12)/polyketone (PK) blends, with the corresponding distribution of PK domain size shown in insets. (**a**) A8K2; (**b**) A7K3; (**c**) A6K4; (**d**) A5K5.

Figure 1 shows the SEM images of the PA12/PK blends with different compositions. The dispersed PK phase was etched out (see Experimental Section); the size distribution of the dispersed PK domains based on image analysis is shown in Figure 1. It is interesting that a nano-scale droplet morphology was observed when the content of PK is low (Figure 1a). The PK phase was well dispersed in the PA12 matrix with a roughly spherical shape, and the average PK domain size was smaller than 100 nm. With increasing content of PK (Figure 1b,c), PK domains with irregular shapes, larger size and larger size distribution were observed owing to the increased number of droplets and the droplet coalescence. When the content of PK was greater than 40 vol %, large domains (greater than 300 nm) were observed. For sample A5K5, a more complicated morphology was observed, and the PK phase seemed to present a partially continuous state with reversed PA12 droplets in the PK domains. The domain size and size distribution were hard to establish (Figure 1d).

**Figure 2:**
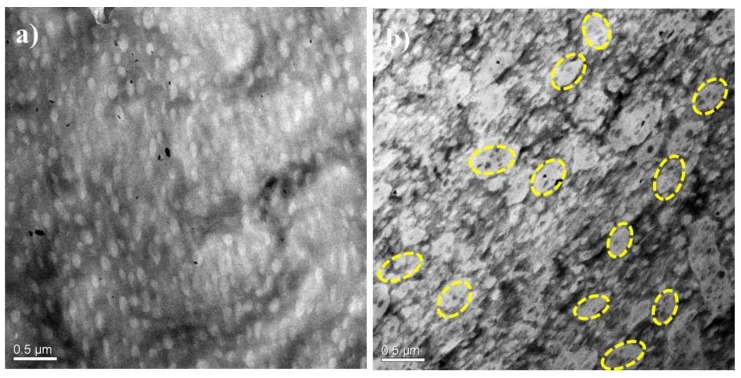
Transmission electron microscope (TEM) images of the PA12/PK blends (**a**) A8K2 and (**b**) A5K5. The bright area is the PK phase.

The morphology of the PA12/PK blends was further examined by TEM; the results are shown in Figure 2. Before characterization, the samples were stained by phosphotungstic acid. In general, the TEM results were consistent with the SEM observations. At low content of PK, isolated, sphere-like domains of PK are seen in Figure 2a. Interestingly, at higher PK concentrations, PA12 droplets were found in larger domains of the PK phase (a domain-in-domain structure, seen in the marked cycle regions of Figure 2b). The average size of PK-rich domains was about 450 nm, which is consistent with the SEM results. The PA12 droplets inside PK-rich domains were about 60 nm. This domain-in-domain fine structure has also been observed in some other blends [32,33].

**Figure 3:**
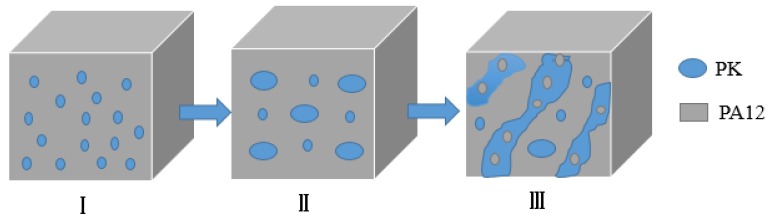
The morphology evolution of the PA12/PK blends on increasing content of PK. (**I**) Nano-dispersion of PK in PA12 matrix; (**II**) submicron- and nanoscale dispersion of PK in PA12 matrix; (**III**) nano-scale domain-in-domain morphology.

Figure 3 schematically illustrates the morphology development of the PA12/PK blends with increasing content of PK, based on the SEM and TEM results. At low contents of PK, nano-sized isolated PK domains formed in the PA12 matrix (see Figure 3I). As the content of PK increased, the number of dispersed droplets, as well as the chance of interaction and coalescence between PK phases, increased. As a result, both submacro- and nano-scale PK dispersed domains were present (Figure 3II). When the content of PK was higher than 40 vol %, some PA12 nano domains dispersed in the merged PK domains to form a special domain-in-domain phase structure.

### 3.2. Miscibility

**Figure 4:**
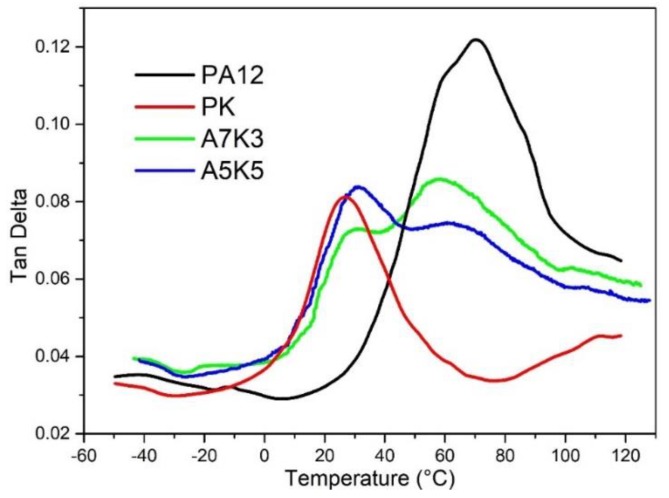
Loss tanδ vs. temperature for pure PA12, PK and blends of A5K5, A7K3.

The glass transition of polymers is due to segmental motion of polymer chains (α or β relaxation, for example). For polymer blends, the glass transition is influenced by the characteristic relaxation of components and their interactions [34]. Therefore, glass transition temperature ($T_{g}$) can be used to characterize the miscibility of the multiple components in a blend [35]. In Figure 4, dynamic mechanical analysis (DMA) of the PA12/PK blends with different compositions is shown. There are two $T_{g}$ peaks in the blends owing to the presence of PA12 ($T_{g}$ = 71 °C) and PK ($T_{g}$ = 26 °C), respectively. The low-temperature peak at 31 °C is due to the motion of the PK segments, and the high-temperature peak at 63 °C is due to the motion of the PA12 segments. The characteristic transition temperatures of the two components shifted toward an intermediate point between 71 and 26 °C, which signifies partial miscibility of the components and is consistent with SEM and TEM results.

**Figure 5:**
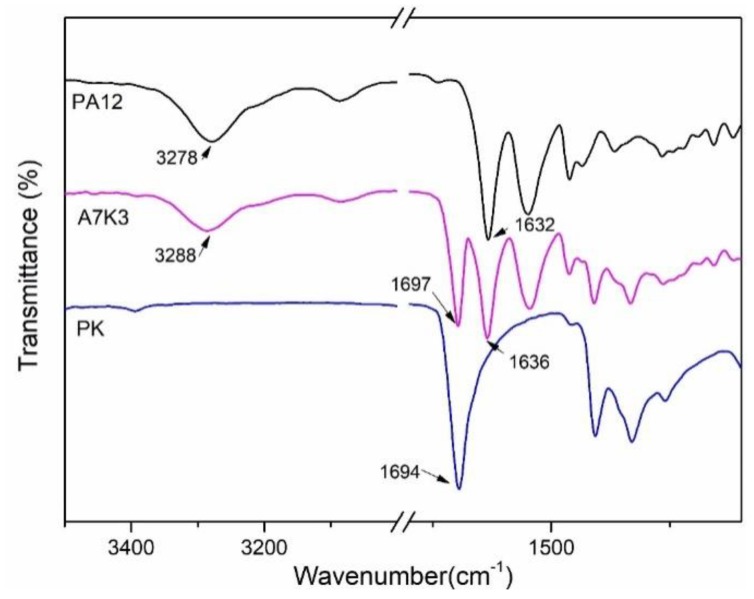
Fourier Transform Infrared Spectroscopy (FTIR) spectra of PA12, PK and the blend A7K3.

For the PA12/PK blends, hydrogen bonds formed between PA12 and PK: the H atoms on the amide groups in PA12 acted as donors and the O atoms on the carbonyl groups in PK acted as acceptors, which played an important role in improving the miscibility of the components, as can be readily found from the FTIR results [36,37]. It has been well documented that the stretching vibration of the C=O bond in PK produces an absorption band at 1694 cm^−1^. For PA12, a stretching vibration of N–H and C=O bonds occurred at 3278 cm^−1^ and 1639 cm^−1^, respectively. Figure 5 shows the FTIR spectra of PA12, PK and a PA12/PK blend (A7K3). It can be seen that the C=O stretching vibration band of PK shifted toward a higher frequency (1697 cm^−1^), whilst the N–H stretching band of PA12 shifted from 3278 to 3288 cm^−1^. This band-shifting provides spectroscopic evidences for the hydrogen bonding interactions, which contributes to the improved miscibility of PA12 and PK in the blends.

It has been widely accepted that the interfacial tension plays an important role in the miscibility, and thus the phase behavior and morphology of polymer blends [38]. For a two-phase system, low interfacial tension often leads to small domains and a narrow size distribution of the dispersed phases. It also helps to form an interfacial layer that can stabilize the phase structures and depress the phase coalescence of the dispersed particles [39].

To describe the interfacial tension in a polymer blend, a simple and convenient model was suggested by Wu et al., which has been used widely [40]. For the calculation of interfacial tension, contact angle measurements were performed to give the surface tension of the PA12/PK blends; the results are shown in Table 1.

Then, according to Wu’s model, for a two-phase system, the overall interfacial tension of the blend is related to the surface tension of the components. The relationship between interfacial tension and surface tension based on the harmonic mean equation is given by Equation (4):

(4) $$\gamma_{12}=\gamma_{1}+\gamma_{2}-4\left[\frac{\gamma_{1}^{d}\gamma_{2}^{d}}{\gamma_{1}^{d}+\gamma_{2}^{d}}+\frac{\gamma_{1}^{p}\gamma_{2}^{p}}{\gamma_{1}^{p}+\gamma_{2}^{p}}\right]$$

where $\gamma_{12}$ is the overall interfacial tension of the blend; $\gamma_{1}$ and $\gamma_{2}$ are the surface energies of component 1 and component 2, respectively; $\gamma_{1}^{d}$ and $\gamma_{2}^{d}$ are the corresponding dispersion components; and $\gamma_{1}^{p}$ and $\gamma_{2}^{p}$ are the polar terms. The $\gamma_{12}$ calculated was 0.70 mN/m, while the $\gamma_{12}$ value for well-known miscible polymer blends, such as high density polyethylene (HDPE) and ethylene-propylene-diene monomer (EPDM), was 0.8 mN/m [41], which suggests that the miscibility between PA12 and PK is quite good.

Therefore, the hydrogen bond interaction and low interfacial tension lead to a good miscibility of the components that plays a crucial role in forming nanostructured PA12/PK blends. The microscopic morphologies and spectroscopic evidences, along with the interfacial tension, suggest the following mechanism, accounting for the phase morphology of the PA12/PK blends. At the molecular level, hydrogen bonding between PA12 and PK enhances their miscibility, and thus stable phase structure can be produced following the phase separation, which is thermodynamically controlled. During polymer processing, the high shear rate during melt compounding leads to small domain sizes of the dispersed phase, which is kinetics-controlled. Moreover, the low interfacial tension leads to stable interfacial layer and suppresses the coalescence of the dispersed domains. The combination of these factors explains the formation of the nano-structure of the blends.

### 3.3. Crystallization and Melting Behavior

**Figure 6:**
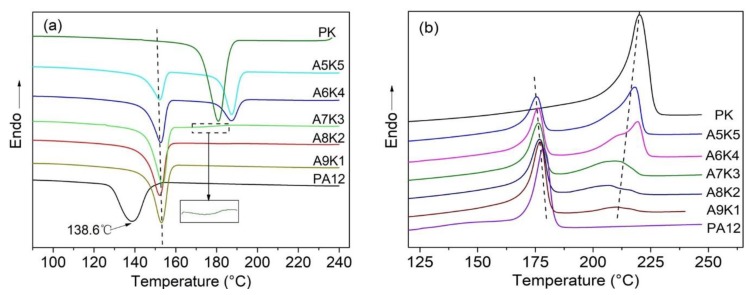
Differential scanning calorimeter (DSC) curves for PA12, PK and the blends. (**a**) Cooling curves (cooling rate: 10 °C/min). The insert shows a magnified fragment of curve for A7K3. (**b**) 2nd cycle heating curves (heating rate: 10 °C/min).

Both PA12 and PK are semi-crystalline polymers, and their crystallization behaviors in the blends were expected to influence the performance of the blends greatly. DSC was used to investigate the melting and crystallization behavior of the PA12/PK blends with different content of PK. The blends were heated up to 250 °C at 10 °C/min under a nitrogen atmosphere and held for 3 min to erase the thermal history and residual stress. The crystallization process was then studied by tracing the endothermic curves during cooling at 10 °C/min, as shown in Figure 6a.

**Figure 7:**
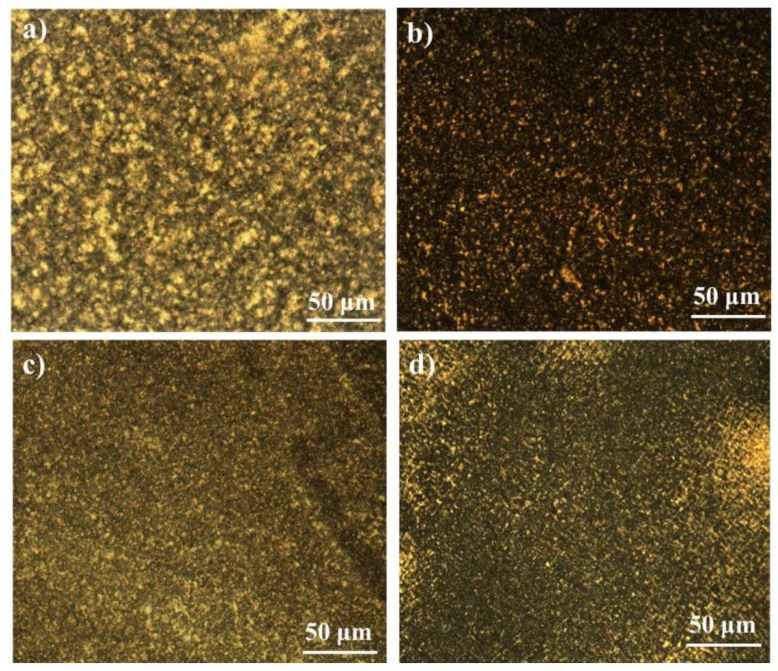
Polarized optical microscopy (POM) images for neat PA12, PK and their blends: (**a**) PA12; (**b**) A8K2; (**c**) A5K5; (**d**) PK.

The crystallization temperatures ($T_{c}$) of pure PA12 and PK are 138.6 and 180.9 °C while the melting points ($T_{m}$) are 178.4 °C and 220.2 °C, respectively. With the introduction of a small amount of PK into PA12, the $T_{c}$ of PA12 increased by approximately 15 °C (see Figure 6a and Table 2), while the crystallization peak of PK was not observed when the content of PK was lower than 20%. At relatively low concentrations, PK dispersed as nanoscale domains, and thus the crystallization of PK was significantly confined and depressed. This confined or fractioned crystallization is a well-known phenomenon in a droplet structure blend, especially when the semi-crystalline dispersed phase size is sufficiently small [42,43]. At the same time, these dispersed PK domains might be able to serve as nucleation sites for PA12 crystallization owing to its higher $T_{c}$ and their hydrogen bonding interactions, which leads to the increase of the crystallization temperature of PA12. The widths of the crystallization peaks in DSC traces are related to the crystallite size distribution in polymer crystals [44], which can be quantified by using the full width at half maximum (FWHM, denoted as ΔW). As seen in Table 2, ΔW values for PA12 in the blends decreased nearly by half when compared to that of pure PA12, which indicates the possible nucleation ability of the homogenously dispersed nanoscale domains of PK for PA12. The strong interface hydrogen interaction between PK and PA12 lead to a low crystallinity of both polymers. Polarized optical microscopy (POM) observations showed that for the PA12/PK blends with low PK content, much smaller spherulites formed as compared to pure PA12 (seen in Figure 7a,b). All these observations consistently indicate the homogenously heterogeneous nucleation of PK for the crystallization of PA12 in the PA12/PK blends.

When further increasing the content of PK, the crystallization behavior of PA12 showed a similar trend; however, the crystallinity was significantly depressed, most likely due to great interface molecular chain absorption and the change of the morphology of the blends. At the same time, the crystallization peak of PK started to show up. It was found that for PK, the $T_{c}$ increased and ΔW narrowed compared to those of pure PK when the content of PK was up to 40%, indicating that the crystallization of PK was also kinetically accelerated by the hydrogen bonding interaction between the two components.

The melting behaviors of the PA12, PK and PA12/PK blends are illustrated in Figure 6b and the derived parameters are included in Table 3. Through the subsequent melting curve and the parameters, we found that there are still melting peaks of PK in A8K2 and A9K1 blends; however, there was no cold crystallization phenomenon. Thus, PK might crystallize at a lower temperature and the crystallization peaks overlap with that of PA12. The melting temperature, $T_{m}$, of a semi-crystalline polymer is related to the lamellar thickness of the crystals. For the PA12/PK blends, a general trend was that $T_{m}$ for the PA12 and PK in the blends were systematically lower than those for the homopolymer counterparts. For example, $T_{m}$ of pure PA12 decreased from 178.4 °C for the homopolymer to 175.6 °C in the A5K5 blend. Similarly, $T_{m}$ of pure PK was 220.2 °C and decreased to 210.0 °C for the A9K1 blend. These results are consistent with the DSC traces in the cooling process and the SEM, TEM and POM results. The morphological evidences and thermal analysis suggested that the confinement effect, that is the crystallization in the phase structures in the blends, along with the hydrogen bonding interaction of the components played an important role in thermodynamic properties of the PA12 and PK components in the blends. Whilst the crystallization kinetics was enhanced (e.g., crystallization takes place at higher temperatures), the crystals were thermodynamically less stable (e.g., $T_{m}$ decreases).

To clarify the structural evolution of PA12 and PK in the blends during crystallization, in situ WAXD was performed. The blend samples (with different content of PK) were heated up to 240 °C and were held at 240 °C for 3 min before cooling down. The diffraction patterns for the samples at 240 °C show only amorphous halos, indicating that PA12 and PK crystals have been melted out. The samples were then cooled down to two temperatures, namely, 170 and 25 °C, at the rate of 10 °C/min, and the diffraction patterns were collected.

**Figure 8:**
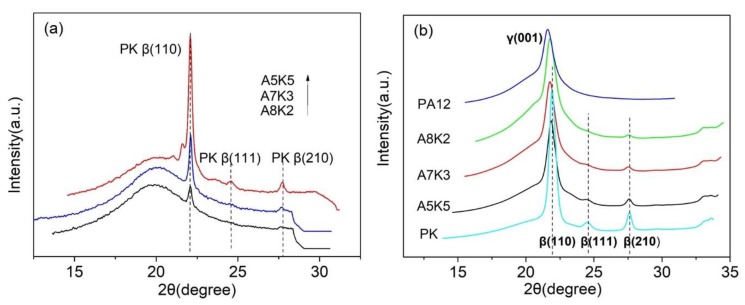
One-dimensional (1D)-wide angle X-ray (WAXD) intensity curves of A5K5, A7A3 and A8K2 blends: (**a**) at 170 °C; (**b**) at 25 °C.

Figure 8 shows the one-dimensional (1D) diffraction profiles of the samples at 170 and 25 °C. It can be seen that PK crystallized in the β-form, showing three characteristic Bragg peaks at 22° (100), 24.8° (111) and 28° (210) [45]. PA12 showed only one characteristic peak at 21.4, due to (001) of the γ-phase [46]. At 170 °C, the crystallization of PK was observed when its content was higher than 20%. However, at lower concentrations, for example for the sample A8K2, only (110) of β phase was visible, and all high-order Bragg peaks, such as (111) and (210), were absent. This suggests that the crystallinity of PK in the blend was low and the crystals were less ordered. The same trend can be observed for the data at 25 °C as shown in Figure 8b. For pure PK, (111) and (210) planes were clearly visible, however, they became weak when the content of PK in the blend was low. It is known that disorder effects, both the first and second kinds, can smear Bragg’s peaks, with the former being able to suppress peak intensity and the latter the breadth [47]. The PK chains in the blend, owing to the strong hydrogen bonding interactions with PA12 chains, were disturbed and crystallized in a less ordered form, and such an effect became severe when a blend was largely composed of PA12. Moreover, as can be seen from Figure 8, the widths of the Braggs peaks of PK changed with its content. When PK content was low, the Braggs peaks seemed to be broader. At a low-angle (take (110) for example), this broadening decreased severely, owing to the disorder effects and the grain size of polycrystallites. Therefore, the WAXD data provide structural support to the DSC results, that is, a lower content of PK forms nano-sized domains in the blends, which not only suppresses the crystal order, but also limits the crystal size of PA12.

Thus, the microscopic, spectroscopic, calorimetric and diffraction results complement each other showing the molecular interaction between PK and PA12 and the confinement effect on the crystallization of both components in the PA12/PK blends.

### 3.4. Toughness

**Figure 9:**
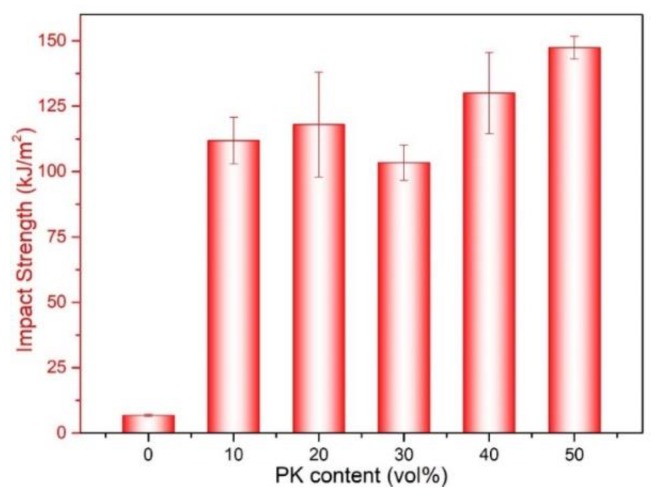
The impact strength of PA12 and the PA12/PK blends.

The impact strength of neat PA12 and PA12/PK blends with different PK content are shown in Figure 9. The impact strength of the PA12/PK blends was significantly enhanced compared to that of PA12. For instance, with the introduction of 10 vol % PK, the impact strength of the blend increased from 6.8 to 111.9 kJ/m^2^ (about 16 times higher than that of pure PA12). However, for the blend with 30 vol % of PK, the impact strength slightly decreased. This might be related to the phase morphology of the blends (morphology II, submicro domain size, as can be seen in Figure 1 and Figure 3). The increased domain size decreased the surface areas of the dispersed phase, which caused a drop in the mechanical performance. After that, the impact strength of the blends became higher again if the PK content was above 40 vol % (e.g., A6K4 and K5A5).

**Figure 10:**
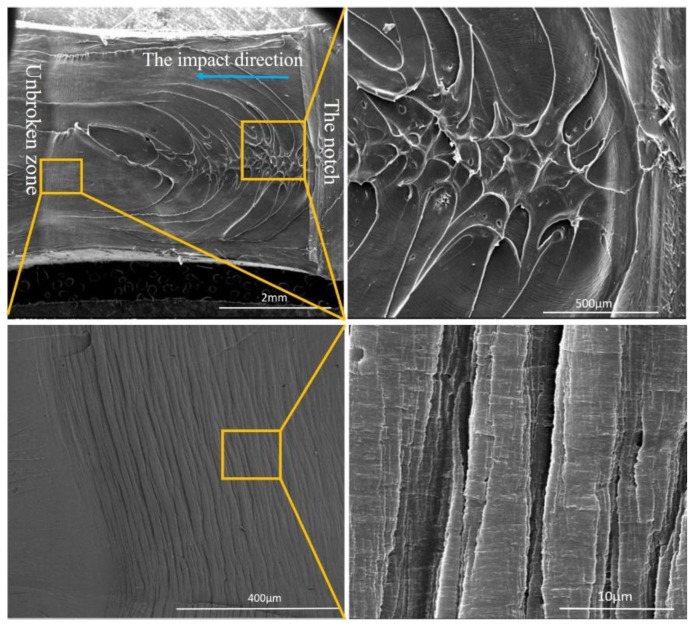
SEM images of impacted surface of the A8K2 blend.

It is hard to explain the toughening mechanism of the PA12/PK blends accurately and comprehensively through the rigid organic filler (ROF) toughening mechanism, which is one of the few mechanisms generally used in rigid plastics/plastics system [48]. However, the ROF toughening works mainly by the deformation (cold drawing) of the dispersed brittle plastics particles in ductile plastic matrix. From SEM images of fracture surface after impact test (Figure 10), an obvious plastic deformation region and shear was observed instead of a deformation of PK particles. Thus, the ROF toughening mechanism could not explain the superior toughness of the PA12/PK blends alone. Here, we try to illustrate the superior toughness of the PA12/PK blends from the following aspects.

Firstly, the change of crystalline structures contributed to the toughening effect. It has been revealed that the number of PA12 crystals increased greatly and the spherical size reduced obviously in the blends (seen from Figure 7). Generally, the toughness of the semi-crystalline polymer with smaller spherulites is higher than that of polymers with larger crystals [49,50]. At the same time, the crystallinity and crystal size of PK decrease greatly due to the hydrogen bonding interaction and the morphologically confined crystallization, which lead to a lower modulus. Thus, crazing and shear yielding are more prone to occur to dissipate greater energy during impact fracture.

Secondly, when nano-sized PK domains are formed (essentially at low PK contents) while the nanodomains are well dispersed, they can cause great plastic deformation in the matrix under impact [51]. Wu [52] rationalized that in this case the impact energy is mainly dissipated in the plastic deformation process, as considerable number of plastic deformation regions can be clearly observed in Figure 10. This is also consistent with the results from Li et al., observed in the nanostructured PVDF/ACM blends [22].

Finally, the nanostructured morphology generates a high surface area, which in return promotes the interaction between PA12 and PK (essentially hydrogen bonding). The formed fine interfacial layers could enhance the interfacial adhesion and facilitate stress dissipation between the phases. In the impact test, the PK dispersed domain tended to deform or lead cavities to absorb the impact energy. Therefore, the nanostructured morphology and hydrogen bonding interaction are essential factors for the superior toughness of the blends.

## 4. Conclusions

The morphology and crystallization of the PA12/PK blends showing greatly improved impact toughness were examined. The results demonstrated that PA12 and PK show intrinsic partial miscibility due to hydrogen bonding between the two polymers, which produces interesting phase morphologies depending on the PA12/PK ratio. When the content of PK in the blend was low, nano-sized isolated PK domains formed in PA12 matrix; the domain size increased as the PK content became higher. When the PK concentration exceeded 40 vol %, domain-in-domain morphologies were observed in the blends. The crystallization of both PA12 and PK components were accelerated, however, and resulted in less stable crystals according to the DSC and WAXD studies. The impact tests indicated that the PA12/PK blends showed significantly improved impact strength owing to the nanostructured morphology, interfacial hydrogen bonding and confined crystallization behaviors of the nanoscale domains. Our results bring new insights into the structure-property relationship of the nano-structured polymer blends.

## Figures and Tables

**Table 1:** Contact angle and surface tension of PA12 and PK.

Sample	Contact Angle (°)		Surface Tension (mN/m)		
	Water	Diiodomethane	Total (*γ*)	Dispersion Component (*γ^d^*)	Polar Component (*γ^p^*)
PA12	81.1	41.2	39.96	36.14	3.83
PK	73.8	23.7	47.94	42.96	4.99

**Table 2:** Crystallization parameters of PA12, PK and the blends determined from Figure 6a.

Samples	PA12				PK			
	*T_c_* (°C)	Tonset (°C)	ΔW (°C)	ΔHc (J/g)	*T_c_* (°C)	Tonset (°C)	ΔW (°C)	ΔHc (J/g)
PK	-	-	-	-	180.9	186.5	8.4	78.5
A5K5	152.1	155.6	6.6	21.2	187.4	191.7	6.1	32.7
A6K4	152.6	156.1	6.5	33.0	187.3	192.3	7.2	18.2
A7K3	153.5	157.1	6.6	43.3	175.8	187.3	17.7	3.8
A8K2	152.2	157.0	6.8	46.0	-	-	-	-
A9K1	153.1	157.3	6.7	47.8	-	-	-	-
PA12	138.6	147.3	12.0	49.7	-	-	-	-

**Table 3:** Melting parameters of PA12, PK and the blends determined from Figure 6b. *Xc* are normalized.

Samples	PA12			PK		
	*T_m_* (°C)	*ΔH_m_* (J/g)	*X_c_* (%)	*T_m_* (°C)	*ΔH_m_* (J/g)	*Xc* (%)
PK	-	-	-	220.2	86.85	39.1
A5K5	175.6	13.58	14.8	218.3	34.16	27.3
A6K4	176.2	18.09	15.7	219.4	26.91	26.3
A7K3	177.2	21.82	15.9	209.7	14.75	18.8
A8K2	176.8	30.24	18.9	207.0	7.74	14.5
A9K1	177.1	40.5	21.9	210.0	4.24	15.6
PA12	178.4	45.66	21.8	-	-	-
